# Lipid metabolites as biomarkers and therapeutic targets in oral squamous cell carcinoma

**DOI:** 10.1186/s12903-025-06700-0

**Published:** 2025-08-31

**Authors:** Hexin Ma, Chang Liu, Xibo Li, Lihua Zuo, Chunshen Li, Xiaohui Xu, Shilong Zhang, Xiang Ma, Erli Yue, Bin Qiao, Yifei Wang, Wantao Chen, Zhi Sun, Hongyu Zhao

**Affiliations:** 1 The First Affiliated Hospital of Zhengzhou University, Zhengzhou, 450000 China; 2 School and Hospital of Stomatology of Zhengzhou University, Zhengzhou, 450000 China; 3 Department of Pharmacy, The First Affiliated Hospital of Zhengzhou University, Zhengzhou, 450000 China; 4 Henan Engineering Research Center of Clinical Mass Spectrometry for Precision Medicine, Zhengzhou, 450000 China; 5 Department of Stomatology, The First Affiliated Hospital of Xi’an Jiaotong University, Xi’an, 710061 China; 6 Guangzhou Jinan Biomedicine Research and Development Center Jinan University, Guangzhou, 510632 China; 7 Department of Oral and Maxillofacial-Head & Neck Oncology, Shanghai Ninth People’s Hospital, Shanghai, 200000 China

**Keywords:** Non-target lipidomics, Biomarkers, Transcriptomics, DGKG

## Abstract

This study explores the association of lipid metabolism disruption and Oral Squamous Cell Carcinoma (OSCC). We aim to identify specific lipid biomarkers and therapeutic targets for OSCC. We included 78 OSCC patients and 80 healthy controls, and applied non-target lipidomics and transcriptomics for comprehensive analysis. Using ultra-high-performance liquid chromatography quadrupole-Orbitrap high-resolution accurate mass spectrometry (UHPLC/Q-Orbitrap HRMS) coupled with machine learning for diagnostic modeling, we identified potential lipid biomarkers. Transcriptomic analysis helped in pinpointing genetic and metabolic targets relevant to lipid metabolism in OSCC. Notably, we observed 70 differential lipid metabolites in the OSCC group, with nine achieving an AUC > 0.95, suggesting high potential as biomarkers. A diagnostic model based on 10 differentiated lipids yielded accuracy rates of 98.2% in a training cohort and 95.7% in a validation cohort. Additionally, the overexpression of DGKG, linked to poor prognosis, was noted to enhance migration and invasion of OSCC cells, marking it a potential target for therapy. This research underscores the critical role of lipid metabolic alterations in OSCC and highlights innovative diagnostic and therapeutic avenues.

**Supplementary Information:**

The online version contains supplementary material available at 10.1186/s12903-025-06700-0.

## Introduction

Oral squamous cell carcinoma (OSCC) is the predominant type of head and neck tumors [1], with a high incidence rate, contributing to approximately 377,713 new cases worldwide in 2020 [2].The 5-year survival rate for OSCC is 60–70%, dropping to 50% with lymph node metastasis [3], and further decreasing for advanced lesions [4]. OSCC is often diagnosed at an advanced stage, contributing to high mortality rates [5]. Current treatments involve surgery, often combined with chemotherapy and radiotherapy in advanced cases [6]. Despite recent advances in screening and targeted therapies, OSCC incidence and mortality remain high, and early detection and treatment remain critical challenges [7]. This underscores the need for further research into OSCC mechanisms, biomarkers, and therapeutic targets to improve patient outcomes.

Metabolic reprogramming, a hallmark of cancer, involves changes in amino acid, lipid, and glucose metabolism [8]. Altered lipid metabolism is a key indicator of tumor metabolic reprogramming [9]. Lipids play critical roles in cell membranes, energy storage, and signaling [10]. Since its introduction by Han XL et al. [11] in 2003, lipidomics has become a key field for identifying diagnostic biomarkers across various diseases, including cardiovascular [12], gastrointestinal [13], and urological diseases [14]. However, lipidomics studies on OSCC remain limited. Our previous research identified significant alterations in lipid metabolites, such as LysoPC (16:0), LysoPC (18:1), and arachidonic acid, in OSCC patients compared to controls, suggesting disrupted lipid metabolism in OSCC [15]. Wang et al. [16] found distinct lipid profiles in OSCC patients, with stage-specific lipid markers. The utilization of the SpiderMass technique by Nina Ogrinc et al. [17] further demonstrated lipid metabolic differences between cancerous and adjacent tissues, with potential surgical applications. These findings underscore the value of lipidomics in OSCC research.While prior lipidomics studies in OSCC primarily focused on single-dimensional analyses—either tissue-level lipid profiling [17] or plasma biomarker discovery [16], our study introduces a three-dimensional innovation: a) Multi-omics integration: First to combine serum lipidomics (LC–MS/MS) with tissue transcriptomics and functional validation, revealing systemic-to-cellular metabolic cascades; b) Identification of DGKG as a central regulator bridging lipid reprogramming and tumor metastasis; c) We establish a serum-based machine learning classifier that achieves 95.7% accuracy in distinguishing OSCC, with potential value for screening high-risk individuals (e.g., those with oral potentially malignant disorders such as leukoplakia, whose malignant transformation rate ranges from 1.1% to 40.8% [18]."

In light of the burgeoning research on lipid metabolism in cancer, scholars have increasingly focused on investigating the lipid metabolism-related mechanisms of OSCC. Hu et al. [19] reported that obesity is linked to poor outcomes in early-stage (T1/2N0M0) OSCC patients. In the tumor microenvironment, dysregulation of obesity-related genes and lipid signaling disrupts lipid metabolism, promoting tumor growth [20–22]. Subhayan Sur et al. [23] found that bitter melon extract (BME) reduced phosphatidylcholine (PC) and phosphatidylethanolamine (PE) species in OSCC cell lines, impairing lipogenesis and inhibiting cell proliferation. Additionally, a high-fat diet in an OSCC mouse model promoted tumor development by increasing myeloid-derived suppressor cells and altering the local microenvironment [24]. These findings highlight the importance of further research into OSCC lipid metabolism to identify biomarkers and therapeutic targets.

Diacylglycerol kinase γ (DGKG), part of the DGK family with 10 isozymes, catalyzes the conversion of diacylglycerol to phosphatidic acid, playing a key role in cellular signaling. Despite its importance, DGKG's role in cancer remains underexplored [25]. Diacylglycerol acts as a lipid second messenger, activating proteins like protein kinase C and Ras guanosine nucleotide-releasing proteins, while phosphatidic acid regulates various signaling pathways [26]. Diacylglycerol acts as a lipid second messenger, activating proteins like protein kinase C and Ras guanosine nucleotide-releasing proteins, while phosphatidic acid regulates various signaling pathways and in colorectal cancer, epigenetic silencing of DGKG contributes to tumorigenesis [27]. In OSCC, increased DGKG expression is linked to patient prognosis, suggesting its potential as a therapeutic target.

**Fig. 1:**
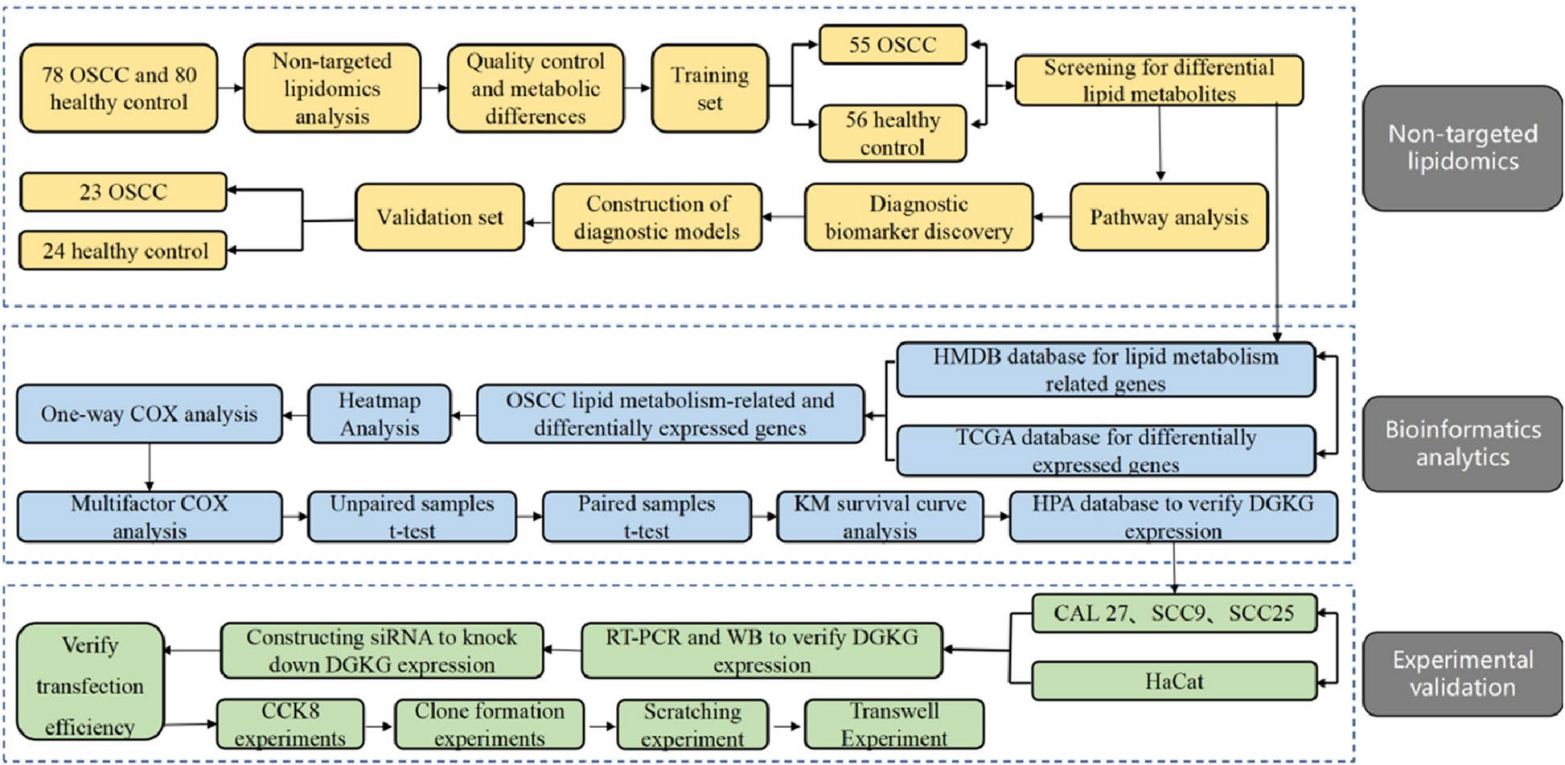
Study design and workflow

This project aims to use liquid chromatography-mass spectrometry combined with non-targeted lipidomics to profile lipid metabolism in OSCC and identify potential diagnostic biomarkers for early detection models. Additionally, transcriptomics data will be integrated to identify lipid metabolism-related differentially expressed genes, including DGKG, and verify its expression in OSCC tissues and cell lines. Molecular biology experiments will assess the impact of DGKG on OSCC cell functions. This study offers new insights for OSCC diagnosis and potential therapeutic targets. The study procedure is shown in Fig. 1.

## Materials and methods

### Research subjects

This study included 78 OSCC patients from the Department of Oral and Maxillofacial Surgery at the First Affiliated Hospital of Zhengzhou University (August 2019—January 2022) and 80 healthy individuals from the Medical Examination Department. Sample sizes in lipidomics studies often vary depending on study objectives (discovery vs. validation), platform sensitivity, and biological variability. Our cohort (*N* = 80 per group) is consistent with or exceeds those in comparable studies [28, 29], where group sizes ranged from 30–80. Additionally, power analysis based on pilot data for key discriminatory lipids (LysoPC(16:0)) indicated that a minimum of 68 samples per group was required to detect a large effect size (Cohen’s d = 0.8) with 80% power. We enrolled slightly more participants (78 OSCC and 80 controls) to account for experimental variation. Blood samples and clinical data were collected from all participants. The study was approved by the Ethics Committee of the First Affiliated Hospital of Zhengzhou University (approval number: 2020-KY-036), with informed consent obtained in accordance with the Declaration of Helsinki. Patients were randomly divided into a training set (55 OSCC patients, 56 healthy controls) and a validation set (23 OSCC patients, 24 healthy controls) in a 7:3 ratio. Inclusion criteria required pathologically confirmed primary OSCC, no prior surgery or chemotherapy, and informed consent. Exclusion criteria included hyperlipidemia, hypertension, metabolic diseases, and recent severe trauma or surgery. The control group was age-matched and free of metabolic diseases or cancer. Blood samples were collected between 6:00 am and 9:00 am, processed immediately, with serum stored at −80 °C for further analysis.

### Lipidomics analysis

#### Sample pre-processing

Lipid extraction followed published MTBE/methanol protocol [30]. Full reagent volumes and centrifugation parameters are detailed in Supplementary Materials and Methods.

#### Quality control sample preparation

For quality control, 5 µL of each sample was pooled, centrifuged, and the supernatant was collected for mass spectrometry. Six QC samples were tested for stability, and QC samples were inserted every 10 samples during analysis to prevent contamination.

#### UHPLC/Q orbitrap HRMS analysis

Each sample (5 µL) was injected into an ACQUITY UPLC® CSH C18 column at 40 °C. The mobile phases were 10 mM ammonium formate (phase A) and acetonitrile/isopropanol (phase B), with gradient elution from 30 to 100% B. Mass spectra were acquired with HESI in positive and negative modes over a scan range of 80–1200 m/z.

#### Data processing

Mass spectrometry data were processed using Lipid Search v4.0 and normalized to peak area. SIMCA14.1 was used for PCA and OPLS-DA to explore intergroup differences, with a 200 permutation test to prevent overfitting. Metabolites with VIP > 1 were selected, and t-tests identified significant lipid differences based on *P*-value and fold change. MetaboAnalyst was used for visualizations, including volcano and heat maps, ROC curves, AUC calculation, pathway enrichment, and diagnostic model construction using machine learning.

#### Machine learning analysis

To evaluate the diagnostic performance of lipid features, we performed multivariate ROC analysis using a linear support vector machine (SVM) classifier combined with Monte-Carlo cross-validation (MCCV). Feature importance was ranked automatically, and the top features were used to construct models. Model performance was assessed by AUC with 95% confidence intervals. Further methodological details are provided in the Supplementary Materials and Methods.

### Combined analysis of lipidomic data and transcriptomic data

Lipid metabolism genes linked to differential lipid metabolites were identified from the Human Metabolome Database (HMDB). mRNA sequencing data from 330 OSCC and 32 normal tissues were retrieved from The Cancer Genome Atlas (TCGA), including clinical and survival data. Using R language, differentially expressed genes related to lipid metabolism were identified and visualized via heatmaps. Univariate and multivariate COX regression analyses were performed to assess prognostic significance and select independent prognostic genes. Validation of gene expression was performed using the Human Protein Atlas (HPA) database, which contains immunohistochemical staining data for various proteins [31].

### *In-vitro* experimental validation

#### Cell culture and transfection

Human OSCC cell lines (CAL27, SCC-9, SCC-25) were obtained from ATCC, and the HaCaT cell line from Cell Lines Service. CAL27 and HaCaT were cultured in DMEM with 10% FBS, while SCC-9 and SCC-25 used DMEM/F12 with 10% FBS and 800 ng/ml hydrocortisone. Cells were incubated at 37 °C with 5% CO_2_. The siRNA-DGKG was designed and synthesized by GenePharma, Cells were transfected using jetPRIME following the manufacturer’s protocol, and transfection efficiency was confirmed by Western blot after 48 h.

#### RNA extraction and RT-PCR

Total RNA was extracted using Trizol (Thermo, USA), and concentration was measured using NanoDrop. RT-PCR was performed using Takara kits, and GAPDH was used for normalization. The relative gene expression was calculated using the 2-ΔΔCt method.

#### Western blot

Proteins were extracted with RIPA buffer and quantified using the BCA assay. Protein samples (15 µg) were separated by SDS-PAGE and transferred to PVDF membranes. After blocking, membranes were incubated with primary antibodies overnight at 4 °C and secondary antibodies after washing. The antibodies used were DGKG (Abcam, 1:1000) and GAPDH (Proteintech, 1:1000).

#### Cell viability, invasion and migration assay

Cell viability was assessed with CCK8 in 96-well plates at 0, 24, 48, and 72 h post-seeding. Wound healing assays were performed in 6-well plates with a scratch made by a pipette tip, and migration was observed at 16 and 32 h. Transwell assays were used for migration (5 × 10^4^ cells) and invasion (double the number), with cells stained and imaged after 24 h.

### Statistical analysis

Statistical analysis was performed using R and GraphPad Prism9.0. Experiments were conducted in triplicates, with t-tests used for two-group comparisons and one-way ANOVA for multiple groups. Data were expressed as mean ± SEM, with significance levels set at *p < 0.05, **p < 0.01, ***p < 0.001.

## Results

### Population baseline characteristics

A total of 111 participants were included in the training set (55 OSCC patients, 56 healthy controls), and 47 participants in the validation set (23 OSCC patients, 24 healthy controls). No significant differences were found in age, gender, or BMI between OSCC patients and healthy controls in both sets (*P* > 0.05). The mean age of OSCC patients was 50.49 years (± 15.61) in the training set and 49.39 years (± 17.17) in the validation set. However, sleep deprivation and lack of physical activity showed significant differences between OSCC patients and controls in the training set (*P* < 0.05), suggesting potential roles in cancer development. Demographic details are presented in Table 1.

**Table 1:** The baseline characteristics of participants

	Training set		*P* value	Validation set		*P* value
	OSCC (*n* = 55)	HC (*n* = 56)	Training set	OSCC (*n* = 23)	HC (*n* = 24)	Validation set
Gender						
Male	22 (19.8%)	27 (24.3%)	0.50	16 (34%)	19 (40.4%)	0.67
Female	33 (29.7%)	29 (26.1%)		7 (14.9%)	5 (10.6%)	
Age (years)	50.49 ± 15.61	45.54 ± 12.96	0.07	49.39 ± 17.17	43.29 ± 14.46	0.19
BMI (kg/m^2^)	23.31 (20.57,25.19)	22.31 (20.27,24.86)	0.35	24.92 ± 3.07	24.33 ± 2.83	0.50
Smoking history	10 (9%)	7 (6.3%)	0.57	7 (14.9%)	8 (17%)	1.00
Drinking history	8 (7.2%)	15 (13.5%)	0.18	7 (14.9%)	11 (23.4%)	0.43
Lack of exercise	43 (38.7%)	30 (27%)	0.01*	16 (34%)	13 (27.7%)	0.43
Like spicy food	14 (12.7%)	22 (20%)	0.63	9 (19.1%)	11 (23.4%)	0.87
Like hot food	17 (15.3%)	18 (16.2%)	0.12	7 (14.9%)	11 (23.4%)	0.43
Lack of sleep	19 (17.1%)	6 (5.4%)	0.005*	5 (10.6%)	2 (4.3%)	0.25
Clinical Stages						
I	11 (20%)			3 (13%)		
II	19 (34.5%)			7 (30.4%)		
III	15 (27.3%)			6 (26.1%)		
IV	9 (16.4%)			7 (30.4%)		
Unknown	1 (1.8%)					

### Quality control and pattern recognition analysis

**Fig. 2:**
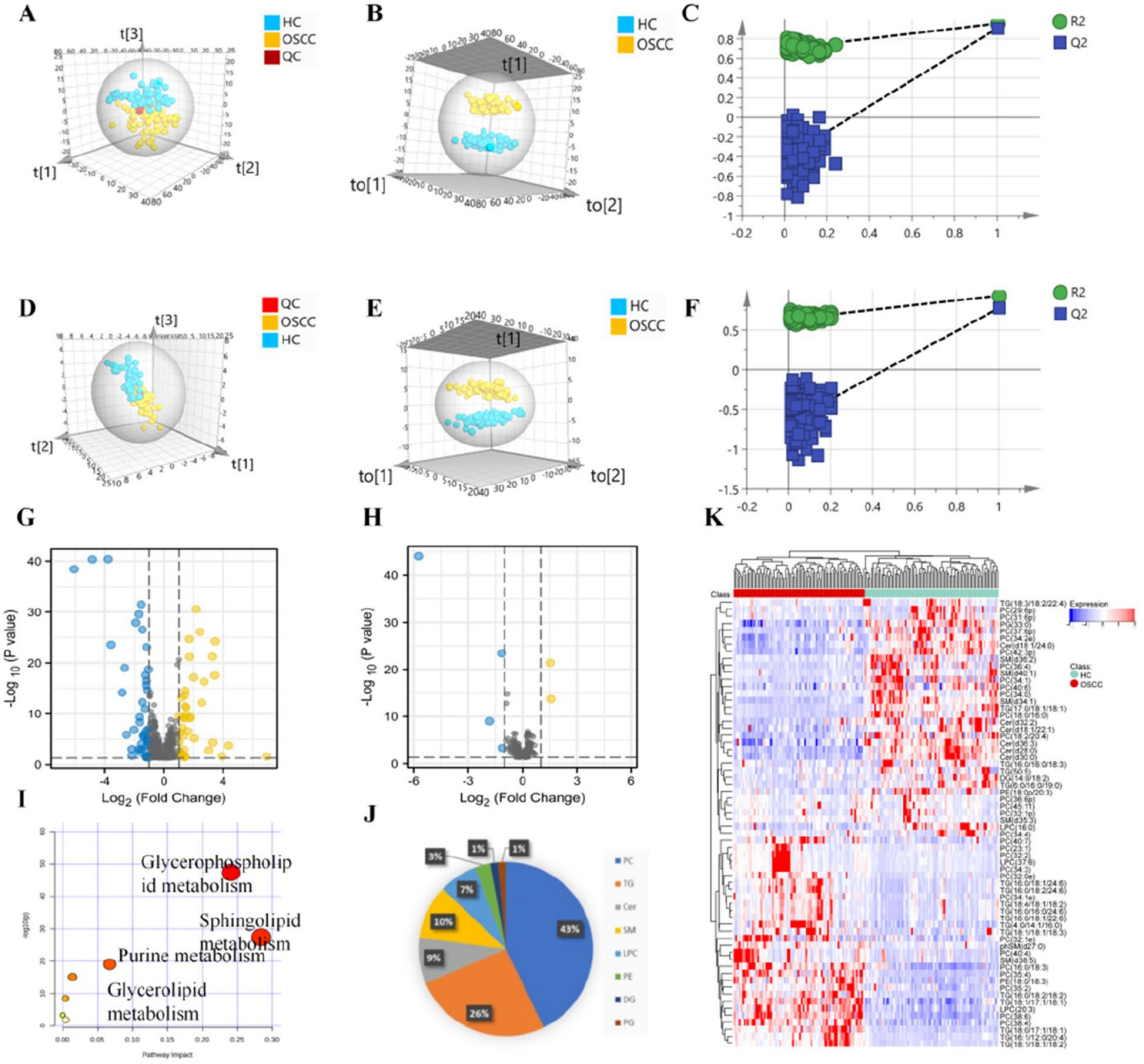
Multivariate statistical analysis and differential lipid metabolite screening between OSCC and healthy control (HC) groups. **A**, **D** PCA score plots showing separation between OSCC and HC groups in positive (**A**) and negative (**D**) ion modes. **B**, **E** OPLS-DA score plots indicating significant discrimination between OSCC and HC samples. **C**, **F** 200-permutation tests confirming the robustness and absence of overfitting in OPLS-DA models. **G**, **H** Volcano plots displaying up- and downregulated lipids in OSCC vs. HC in positive (**G**) and negative (**H**) ion modes (criteria: *P* < 0.05, |log2FC|> 1). **I** Metabolic pathway enrichment analysis of differential lipids, highlighting involvement in glycerophospholipid and sphingolipid metabolism. **J** Lipid class distribution of identified differential metabolites, with PC and TG accounting for 69%. **K** Heatmap of the top differential lipid metabolites between OSCC and HC groups, demonstrating distinct lipid expression patterns

In this study, a total of 78 patients diagnosed with OSCC and 80 healthy individuals were subjected to non-targeted lipidomic analysis. The analysis revealed the detection of 1546 and 611 ion peaks in positive and negative ion mode, respectively, encompassing various lipid classes such as phosphatidylcholine (PC), triglycerides (TG), ceramides (Cer), sphingomyelin (SM), lyso-phosphatidylcholine (LPC), phosphatidylethanolamine (PE), phosphatidylglycerol (PG), among others. The results of principal component analysis (PCA) demonstrated that quality control samples exhibited clustering, suggesting consistent instrument performance and reproducible methodology. Additionally, a clear separation trend was observed between oral squamous cell carcinoma (OSCC) samples and healthy control (HC) samples, indicating significant disparities in lipid metabolism between the two groups (Fig. 2A, D). Subsequently, orthogonal partial least squares discriminant analysis (OPLS-DA) was conducted to identify and distinguish differential lipid metabolites between the two groups.. The results depicted in Fig. 2B and E demonstrate a distinct differentiation between the group with OSCC and the healthy control group, suggesting a perturbation in lipid metabolism within the OSCC group. The OPLS-DA analysis revealed key parameters, including R^2^X, R^2^Y, and Q^2^, with values of 0.509, 0.957, and 0.907 in positive ion mode, and 0.578, 0.931, and 0.774 in negative ion mode, respectively. The values of R^2^Y and Q^2^ exceeding 0.5 indicate stability and reliability of the model. The model was tested for 200 permutations, as shown in the Fig. 2C and F. OPLS-DA in positive ion mode was *R*^2^ = (0.0, 0.708), Q^2^ = (0.0, −0.413); in negative ion mode, *R*^2^ = (0.0, 0.637), Q^2^ = (0.0, −0.667). Both Q^2^ are less than 0, indicating that OPLS-DA had no overfitting occurred and the results were reliable.

### Screening of differential lipid metabolites

Using a significance threshold of *P* < 0.05 and |log2FC|> 1.0, volcano plots (Fig. 2G, B) revealed 39 upregulated and 42 downregulated lipids in OSCC compared to healthy controls (HC) in positive ion mode, and 2 upregulated and 4 downregulated lipids in negative ion mode. After applying VIP > 1, 65 differential lipids were identified in positive ion mode and 5 in negative ion mode, detailed in Table 2. The relative proportions of PC, TG, Cer, SM, LPC, PE, DG, and PG were 43%, 26%, 9%, 10%, 7%, 3%, 1%, and 1%, respectively. The combined PC and TG composition was 69% (Fig. 2J), suggesting a link between disturbances in these lipids and OSCC pathogenesis. Heat map analysis (Fig. 2K) further highlighted lipid level differences between OSCC and HC groups.

**Table 2:** Differential lipid metabolites between the OSCC and the healthy group

No	Lipid name	Ion type	Molecular formula	Class	VIP	FC	*P*-value	Trend	AUC
1	Cer (d28:0)	P	C_28_H_58_O_3_N_1_	Cer	4.74	0.34	3.26E-32	↓	0.982
2	Cer (d30:0)	P	C_30_H_62_O_3_N_1_	Cer	4.63	0.36	2.95E-27	↓	0.969
3	Cer (d32:2)	P	C_32_H_62_O_3_N_1_	Cer	2.39	0.43	2.12E-07	↓	0.760
4	Cer (d36:3)	P	C_36_H_68_O_3_N_1_	Cer	3.81	0.36	5.18E-13	↓	0.947
5	Cer (d18:1/22:1)	P	C_40_H_78_O_3_N_1_	Cer	3.70	0.44	3.57E-17	↓	0.886
6	Cer (d18:1/24:0)	P	C_42_H_84_O_3_N_1_	Cer	2.64	0.41	1.08E-11	↓	0.884
7	DG (14:0/18:2)	P	C_35_H_68_O_5_N_1_	DG	2.29	0.39	4.01E-06	↓	0.729
8	LPC (16:0)	P	C_24_H_51_O_7_N_1_P_1_	LPC	4.32	0.03	4.68E-41	↓	0.979
9	LPC (16:0)	P	C_24_H_51_O_7_N_1_P_1_	LPC	3.05	0.49	2.06E-19	↓	0.856
10	LPC (20:3)	P	C_28_H_53_O_7_N_1_P_1_	LPC	3.11	3.27	2.03E-25	↑	0.873
11	LPC (37:6)	P	C_45_H_81_O_7_N_1_P_1_	LPC	1.23	8.20	1.50E-03	↑	0.512
12	LPC (37:6)	P	C_45_H_81_O_7_N_1_P_1_	LPC	1.58	20.74	1.65E-04	↑	0.635
13	PC (23:1)	P	C_31_H_61_O_8_N_1_P_1_	PC	1.17	2.57	1.19E-03	↑	0.594
14	PC (29:6p)	P	C_37_H_63_O_7_N_1_P_1_	PC	2.05	0.37	1.99E-06	↓	0.708
15	PC (31:6p)	P	C_39_H_67_O_7_N_1_P_1_	PC	2.53	0.32	4.59E-10	↓	0.761
16	PC (32:0e)	P	C_40_H_83_O_7_N_1_P_1_	PC	2.60	5.89	3.26E-32	↑	0.815
17	PC (32:1e)	P	C_40_H_81_O_7_N_1_P_1_	PC	1.67	2.55	2.95E-27	↑	0.693
18	PC (32:1p)	P	C_40_H_79_O_7_N_1_P_1_	PC	2.25	0.50	2.12E-07	↓	0.741
19	PC (32:2)	P	C_40_H_77_O_8_N_1_P_1_	PC	1.53	4.09	5.18E-13	↑	0.598
20	PC (34:0)	P	C_42_H_85_O_8_N_1_P_1_	PC	3.78	0.08	3.57E-17	↓	0.917
21	PC (34:1)	P	C_42_H_83_O_8_N_1_P_1_	PC	1.76	0.22	1.08E-11	↓	0.724
22	PC (34:1e)	P	C_42_H_85_O_7_N_1_P_1_	PC	2.02	2.69	4.01E-06	↑	0.734
23	PC (34:2e)	P	C_42_H_83_O_7_N_1_P_1_	PC	3.48	0.41	4.68E-41	↓	0.903
24	PC (34:3)	P	C_42_H_79_O_8_N_1_P_1_	PC	1.38	9.82	2.06E-19	↑	0.571
25	PC (34:3)	P	C_42_H_79_O_8_N_1_P_1_	PC	3.68	4.49	2.67E-31	↑	0.923
26	PC (34:4)	P	C_42_H_77_O_8_N_1_P_1_	PC	1.63	0.47	5.95E-04	↓	0.654
27	PC (35:2)	P	C_43_H_83_O_8_N_1_P_1_	PC	2.36	2.26	6.97E-14	↑	0.762
28	PC (35:4)	P	C_43_H_79_O_8_N_1_P_1_	PC	3.12	10.73	2.92E-18	↑	0.831
29	PC (36:4)	P	C_44_H_81_O_8_N_1_P_1_	PC	3.26	0.14	6.78E-15	↓	0.837
30	PC (36:6p)	P	C_44_H_77_O_7_N_1_P_1_	PC	2.14	0.40	4.59E-10	↓	0.710
31	PC (37:6p)	P	C_45_H_79_O_7_N_1_P_1_	PC	2.31	0.24	6.35E-13	↓	0.761
32	PC (38:4)	P	C_46_H_85_O_8_N_1_P_1_	PC	3.24	3.31	2.11E-06	↑	0.890
33	PC (38:6)	P	C_46_H_81_O_8_N_1_P_1_	PC	3.62	5.36	9.59E-08	↑	0.956
34	PC (40:4)	P	C_48_H_89_O_8_N_1_P_1_	PC	2.06	3.44	6.73E-06	↑	0.766
35	PC (40:6)	P	C_48_H_85_O_8_N_1_P_1_	PC	4.42	0.01	2.64E-24	↓	0.975
36	PC (40:6)	P	C_48_H_85_O_8_N_1_P_1_	PC	2.29	0.49	2.41E-05	↓	0.811
37	PC (40:7)	P	C_48_H_83_O_8_N_1_P_1_	PC	2.04	2.60	3.26E-12	↑	0.728
38	PC (42:3p)	P	C_50_H_95_O_7_N_1_P_1_	PC	3.56	0.26	2.75E-18	↓	0.946
39	PC (45:11)	P	C_53_H_85_O_8_N_1_P_1_	PC	1.59	0.46	4.41E-05	↓	0.694
40	PE (18:0p/20:3)	P	C_43_H_81_O_7_N_1_P_1_	PE	2.34	0.32	2.67E-31	↓	0.765
41	SM (d34:1)	P	C_39_H_80_O_6_N_2_P_1_	SM	3.37	0.46	2.53E-15	↓	0.864
42	SM (d34:1)	P	C_39_H_80_O_6_N_2_P_1_	SM	4.26	0.31	6.97E-14	↓	0.957
43	SM (d35:3)	P	C_40_H_78_O_6_N_2_P_1_	SM	1.08	0.33	2.92E-18	↓	0.631
44	SM (d36:2)	P	C_41_H_82_O_6_N_2_P_1_	SM	1.82	0.30	6.78E-15	↓	0.734
45	SM (d38:5)	P	C_43_H_80_O_6_N_2_P_1_	SM	2.45	3.32	7.95E-07	↑	0.763
46	SM (d40:1)	P	C_45_H_92_O_6_N_2_P_1_	SM	3.41	0.16	1.43E-07	↓	0.884
47	phSM (d27:0)	P	C_32_H_68_O_7_N_2_P_1_	SM	2.32	0.17	1.28E-06	↓	0.748
48	TG (4:0/14:1/16:0)	P	C_37_H_69_O_6_	TG	1.66	3.09	8.13E-27	↑	0.806
49	TG (6:0/16:0/19:0)	P	C_44_H_85_O_6_	TG	2.67	2.68	4.46E-10	↑	0.808
50	TG (16:1/12:0/20:4)	P	C_51_H_92_O_6_N_1_	TG	2.65	0.43	2.41E-16	↓	0.809
51	TG (16:0/16:0/18:3)	P	C_53_H_100_O_6_N_1_	TG	2.49	3.99	7.43E-18	↑	0.709
52	TG (50:5)	P	C_53_H_96_O_6_N_1_	TG	1.75	0.45	1.70E-05	↓	0.725
53	TG (16:0/18:2/18:2)	P	C_55_H_102_O_6_N_1_	TG	1.83	0.45	1.41E-04	↓	0.833
54	TG (17:0/18:1/18:1)	P	C_56_H_108_O_6_N_1_	TG	2.99	6.57	4.93E-17	↑	0.978
55	TG (18:0/17:1/18:1)	P	C_56_H_108_O_6_N_1_	TG	4.42	0.07	4.14E-41	↓	0.883
56	TG (18:1/17:1/18:1)	P	C_56_H_106_O_6_N_1_	TG	3.38	9.67	5.93E-22	↑	0.890
57	TG (18:1/18:1/18:2)	P	C_57_H_106_O_6_N_1_	TG	2.69	2.69	2.82E-15	↑	0.791
58	TG (18:1/18:1/18:2)	P	C_57_H_106_O_6_N_1_	TG	2.03	2.02	2.89E-10	↑	0.895
59	TG (18:1/18:1/18:3)	P	C_57_H_104_O_6_N_1_	TG	3.21	10.96	5.88E-25	↑	0.650
60	TG (18:4/18:1/18:2)	P	C_57_H_97_O_6_	TG	1.36	2.01	4.44E-05	↑	0.660
61	TG (16:0/16:0/24:6)	P	C_59_H_103_O_6_	TG	1.49	2.11	5.72E-07	↑	0.680
62	TG (16:0/18:1/22:6)	P	C_59_H_101_O_6_	TG	1.67	2.37	5.59E-08	↑	0.702
63	TG (16:0/18:1/24:6)	P	C_61_H_105_O_6_	TG	1.98	2.53	7.03E-10	↑	0.732
64	TG (16:0/18:2/24:6)	P	C_61_H_103_O6	TG	2.15	2.79	5.93E-12	↑	0.720
65	TG (18:3/18:2/22:4)	P	C_61_H_101_O6	TG	2.13	2.84	1.91E-10	↑	0.765
66	PC (18:0/16:0)	N	C_43_H_85_O_10_N_1_P_1_	PC	4.17	0.02	8.81E-45	↓	0.993
67	PC (16:0/18:3)	N	C_43_H_79_O_10_N_1_P_1_	PC	3.49	2.9	3.95E-22	↑	0.866
68	PC (18:2/20:4)	N	C_47_H_81_O_10_N_1_P_1_	PC	4.05	0.28	9.72E-10	↓	0.864
69	PE (18:0/18:3)	N	C_41_H_75_O_8_N_1_P_1_	PE	3.24	3.0	1.89E-14	↑	0.866
70	PG (33:0)	N	C_39_H_76_O_10_N_0_P_1_	PG	4.71	0.45	3.81E-24	↓	0.973

### Differential metabolic pathway analysis

Metabolic pathway analysis of 70 differential lipid metabolites using MetaboAnalyst identified potential pathogenic mechanisms in OSCC. F igure 2I shows the P values and pathway impact values from the enrichment analysis. The most impacted pathways, in descending order, are glycerophospholipid metabolism, sphingolipid metabolism, purine metabolism, and glycerolipid metabolism, indicating a strong association between these pathways and OSCC progression.

Among them, glycerophospholipid metabolism represents a fundamental process for maintaining cellular membrane integrity and function. Glycerophospholipids not only constitute the principal structural components of cellular membranes but also regulate intracellular signaling and inflammatory responses [32]. Consistent with this, previous studies have demonstrated that dysregulation of glycerophospholipid metabolism plays a critical role in the proliferation of esophageal squamous cell carcinoma and breast cancer [33, 34]. Therefore, in the context of OSCC, alterations in glycerophospholipid metabolism may disrupt membrane composition, consequently affecting cellular proliferation and apoptosis.

Similarly, sphingolipid metabolism has emerged as a key pathway in cancer biology. It not only orchestrates the recruitment of immune and non-immune cells within the tumor microenvironment (TME) but also mediates pro-inflammatory signaling cascades. In particular, it facilitates the release of cytokines such as interleukin (IL)−1β, thereby contributing to tumor-promoting inflammation and offering potential targets for therapeutic intervention [35].

### Diagnostic biomarker discovery

**Fig. 3:**
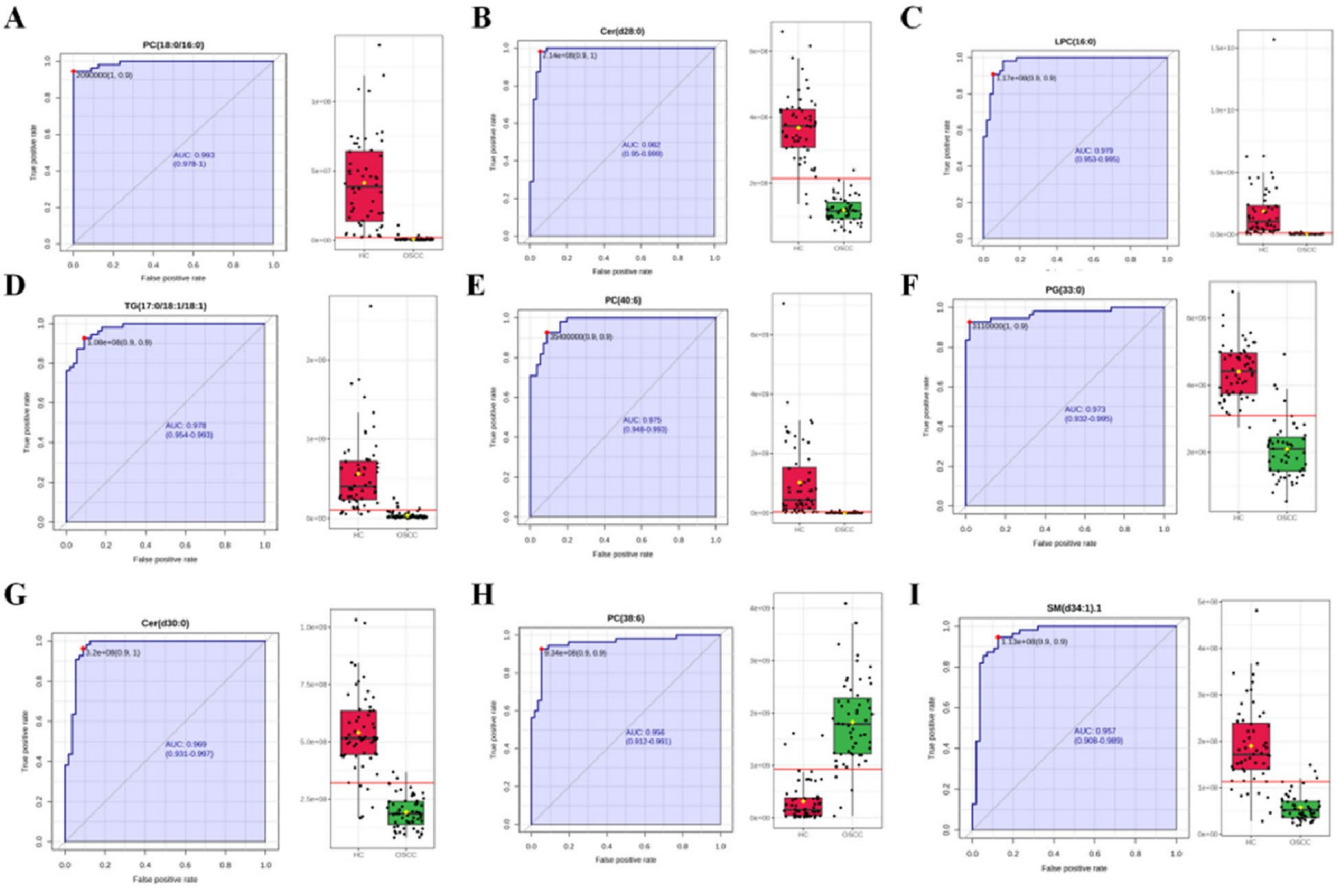
Diagnostic efficacy of lipid biomarkers for OSCC. **A**–**I** ROC curves and AUC values for nine individual lipid metabolites with AUC > 0.95, including PC (18:0/16:0), Cer (d28:0), LPC (16:0), TG (17:0/18:1/18:1), PC (40:6), PG (33:0), Cer (d30:0), PC (38:6), and SM (d34:1)

The identified differential lipids were subjected to single-factor ROC curve analysis using the training set, with AUC values shown in Table 2. Nine metabolites had AUC values greater than 0.95, including PC (18:0/16:0), Cer (d28:0), LPC (16:0), TG (17:0/18:1/18:1), PC (40:6), PG (33:0), Cer (d30:0), PC (38:6), and SM (d34:1). Of these, eight were downregulated and one was upregulated in OSCC. Figure 3 presents the ROC curves, AUC values, and 95% confidence intervals (CI) for the nine metabolites, along with histograms showing content differences between OSCC and healthy controls. These metabolites, individually or combined, could serve as biomarkers for oral cancer detection, with all having AUC values above 0.95, indicating high sensitivity and specificity.

### Diagnostic model construction and validation

**Fig. 4:**
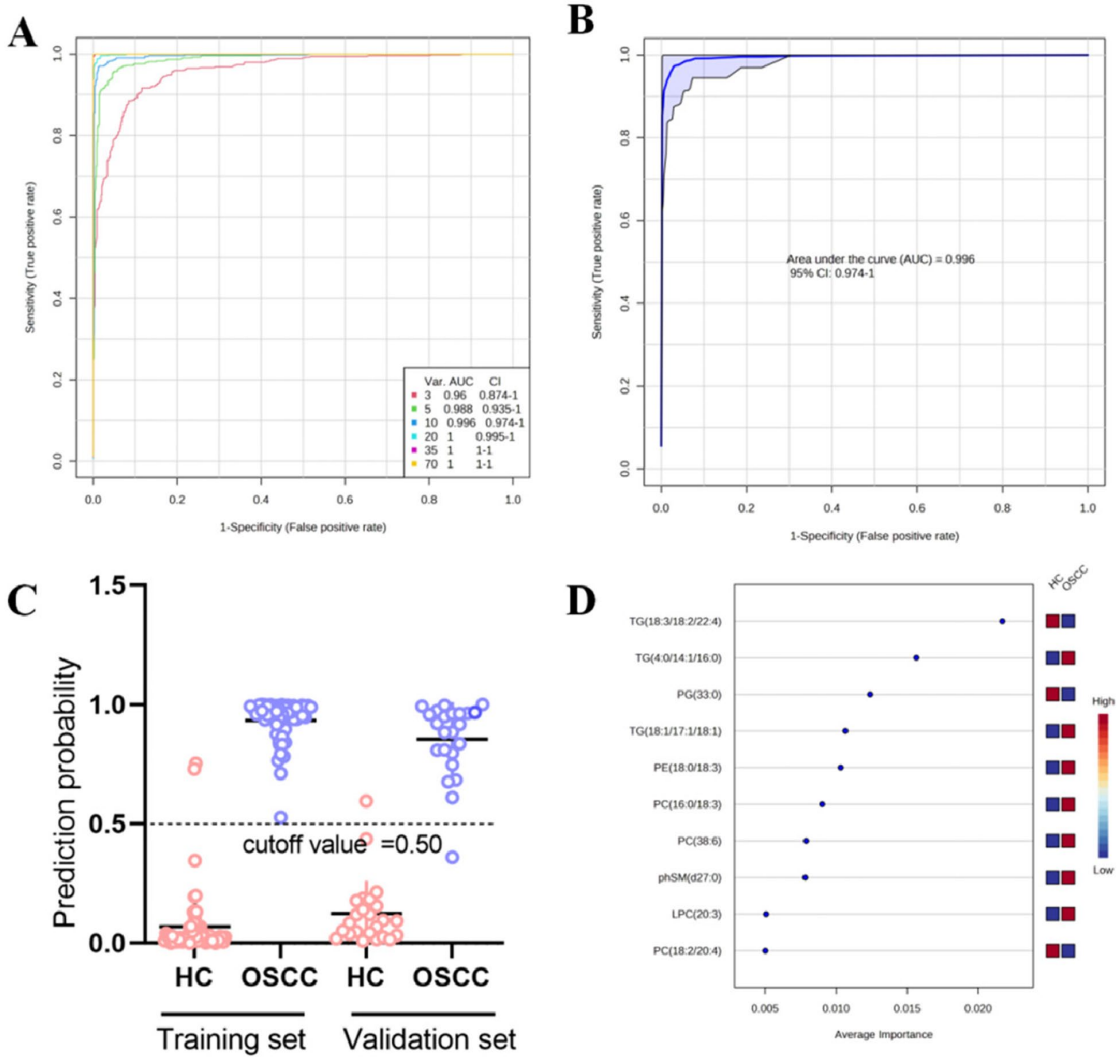
Diagnostic model construction and validation based on lipidomic signatures. **A** Multivariate ROC analysis using SVM and MCCV for models with 3, 5, 10, and 70 lipids; all models show high AUC values. **B** ROC curve for the 10-lipid model with AUC of 0.996 and 95% CI of 0.971–1. **C** Classification results showing diagnostic accuracy of the model in training and validation sets (98.2% and 95.7%, respectively). **D** Top 10 lipid biomarkers selected for diagnostic model construction, ranked by importance

One-way ROC analysis showed that 58 out of 70 lipid metabolites had AUC values above 0.7, indicating high diagnostic accuracy for distinguishing OSCC from healthy controls (HC) in a sample size of 111. However, relying on a single biomarker for a complex disease may reduce accuracy in larger populations. Therefore, multivariate ROC analysis using support vector machines (SVM) and Monte-Carlo cross-validation (MCCV) was conducted (Fig. 4A). The AUC values for models containing 3, 5, 10, and 70 lipids were 0.96, 0.988, 0.996, and 1, respectively. Based on accuracy, cost-effectiveness, and practicality, a diagnostic model using 10 key lipid markers was developed, achieving an AUC of 0.996 (95% CI: 0.971–1) and a diagnostic accuracy rate of 98.2% in the training set (Fig. 4B, C). The top 10 markers included TG (18:3/18:2/22:4), TG (4:0/14:1/16:0), PG (33:0), PC (16:0/18:3), PE (18:0/18:3), and others (Fig. 4D, Table 3). Validation on 47 samples using linear SVM analysis resulted in 95.7% accuracy, with one misdiagnosis among 24 healthy controls and 23 OSCC patients (Fig. 4C).

**Table 3:** The 10 differential lipids that composed the diagnostic model

No	Lipid name	Ion type	Molecular formula	Class	VIP	FC	*P*-value	Trend	AUC
1	PC (34:1)	P	C_42_H_83_O_8_N_1_P_1_	PC	1.76	0.22	1.08E-11	↓	0.724
2	PC (38:6)	P	C_46_H_81_O_8_N_1_P_1_	PC	3.62	5.36	9.59E-08	↑	0.956
3	PC (40:4)	P	C_48_H_89_O_8_N_1_P_1_	PC	2.06	3.44	6.73E-06	↑	0.766
4	phSM(d27:0)	P	C_32_H_68_O_7_N_2_P_1_	SM	2.32	0.17	1.28E-06	↓	0.748
5	TG (4:0/14:1/16:0)	P	C_37_H_69_O_6_	TG	1.66	3.09	8.13E-27	↑	0.806
6	TG (18:1/17:1/18:1)	P	C_56_H_106_O_6_N_1_	TG	3.38	9.67	5.93E-22	↑	0.890
7	TG (18:3/18:2/22:4)	P	C_61_H_101_O6	TG	2.13	2.84	1.91E-10	↑	0.765
8	PC (16:0/18:3)	N	C_43_H_79_O_10_N_1_P_1_	PC	3.49	2.9	3.95E-22	↑	0.866
9	PE (18:0/18:3)	N	C_41_H_75_O_8_N_1_P_1_	PE	3.24	3.0	1.89E-14	↑	0.866
10	PG (33:0)	N	C_39_H_76_O_10_N_0_P_1_	PG	4.71	0.45	3.81E-24	↓	0.973

### Screening of OSCC lipid metabolism-related target genes

**Fig. 5:**
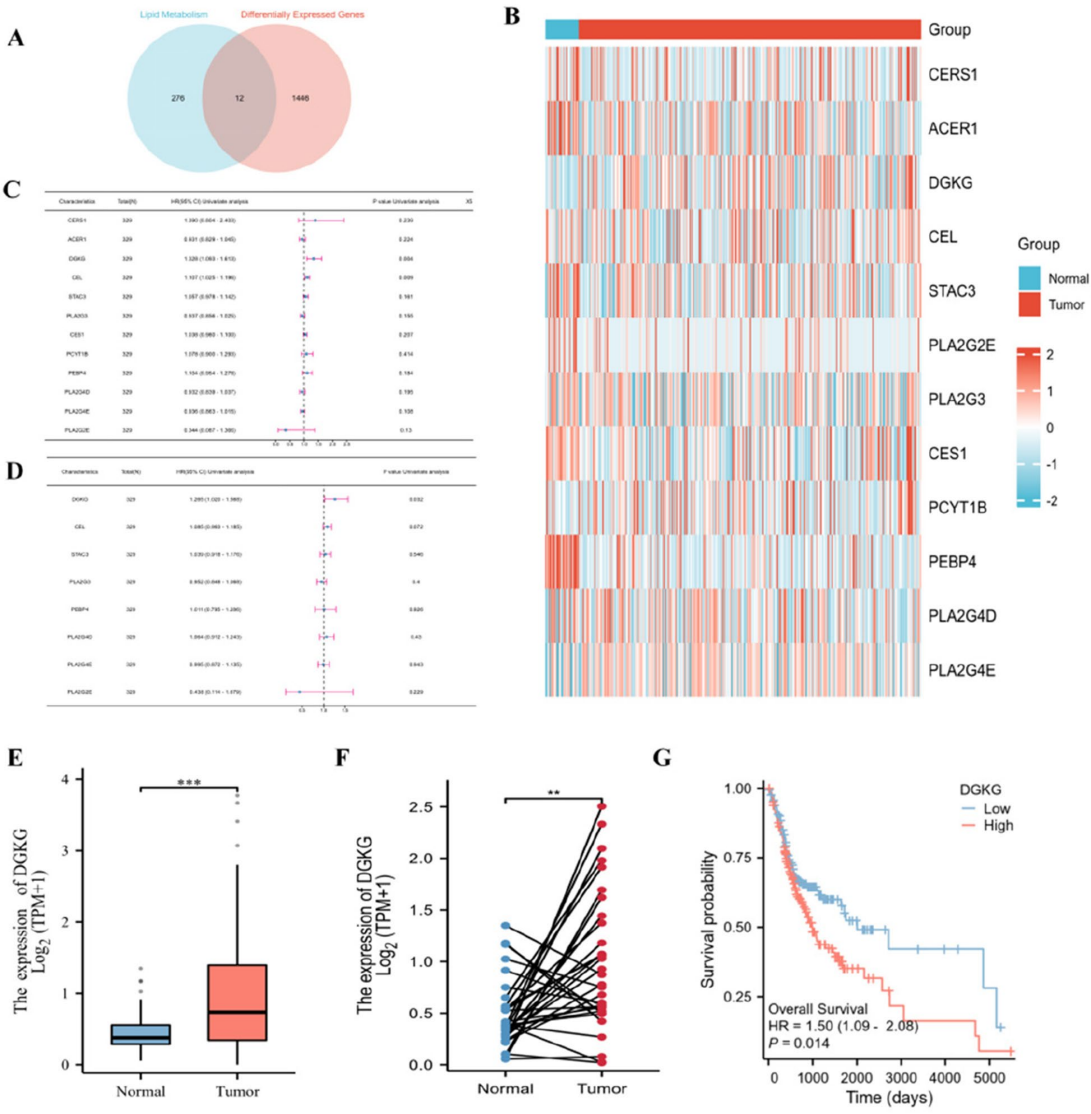
Identification and prognostic analysis of OSCC lipid metabolism–related genes. **A** Venn diagram showing 12 overlapping genes between HMDB lipid-related genes and differentially expressed genes in OSCC. **B** Heatmap showing expression of the 12 genes in OSCC and HC samples. **C** Univariate Cox regression indicating 7 risk and 5 protective genes. **D** Multivariate Cox regression identifying DGKG as an independent prognostic risk gene. **E** Histogram of DGKG mRNA expression in 330 OSCC tissues and 32 paired samples from TCGA. **F** Paired t-test confirming significant differential DGKG expression. **G** Kaplan–Meier survival analysis based on DGKG expression levels

Using the HMDB database, 298 differential lipid-related genes were identified, followed by analysis of differentially expressed genes between OSCC patients and healthy controls using R (Fig. 5A). By intersecting the two sets, 12 common lipid metabolism-related genes were identified. Heat map analysis showed significant differential expression of these genes between OSCC and healthy controls (Fig. 5B). One-way Cox regression revealed that 7 genes had a hazard ratio greater than 1, classifying them as risk genes, while 5 were protective (Fig. 5C). Multivariate Cox regression identified DGKG as an independent risk factor for OSCC (Fig. 5D). A histogram of DGKG mRNA expression in 330 OSCC tissues and 32 paired tissues from the TCGA database showed significant differences (Fig. 5E), confirmed by a paired sample t-test (Fig. 5F). Kaplan–Meier survival analysis demonstrated a clear prognostic difference between high and low DGKG expression groups (Fig. 5G).

### High expression of DGKG in OSCC tissues and cell lines

**Fig. 6:**
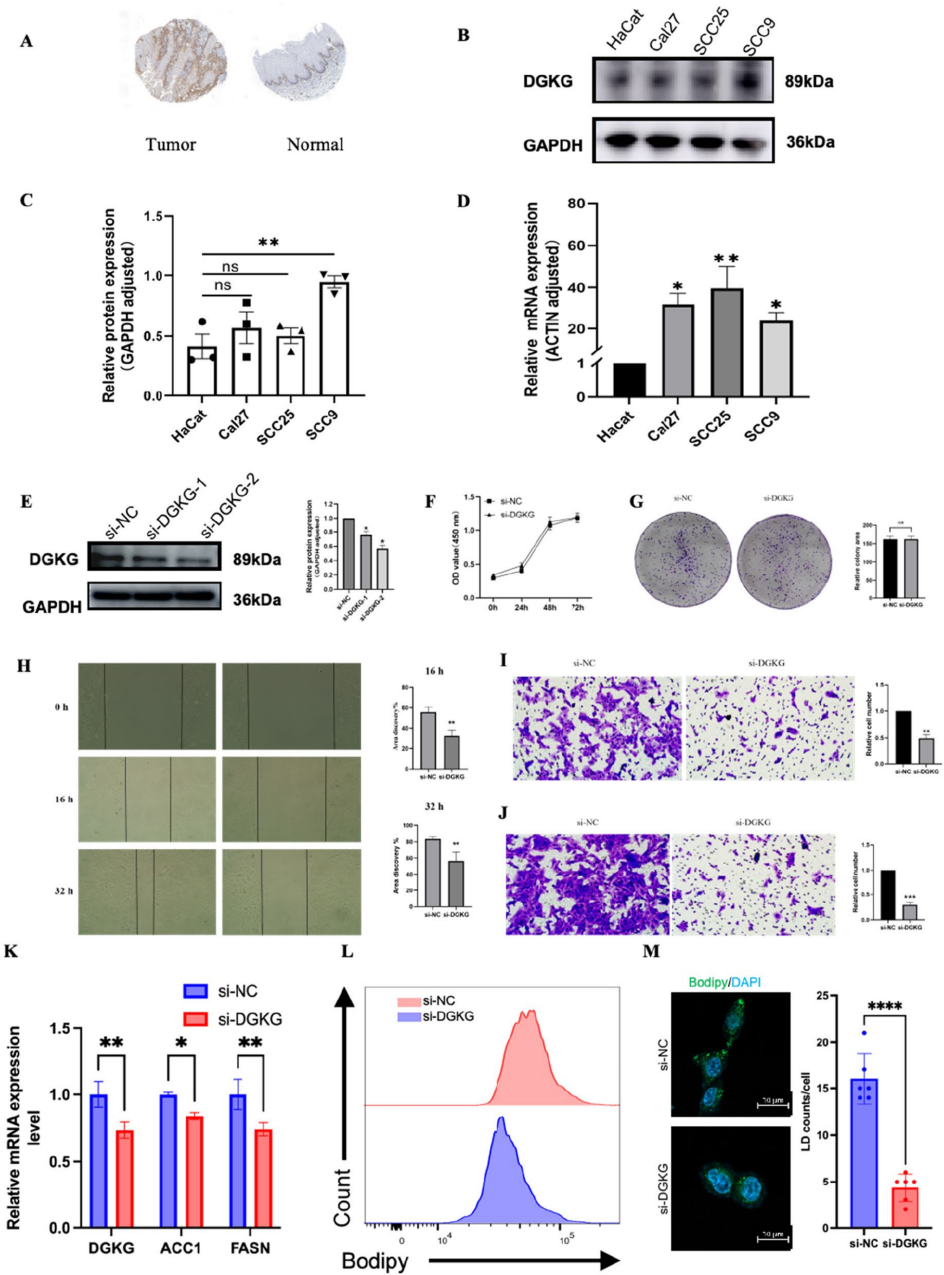
Experimental validation of DGKG expression and functional analysis in OSCC cell lines. **A** HPA database showing increased DGKG protein levels in OSCC tissues. **B** Western blot analysis of DGKG in OSCC cell lines (CAL27, SCC9, SCC25) and control (HaCaT). **C** qRT-PCR analysis of DGKG mRNA levels in cell lines. **D** Quantification of DGKG protein expression. **E** DGKG knockdown efficiency using siRNA; si-DGKG-2 selected for further experiments. **F**, **G** CCK8 and colony formation assays showing no effect of DGKG knockdown on SCC9 proliferation. **H** Wound healing assay showing reduced migration after DGKG knockdown. **I**, **J** Transwell assays demonstrating decreased migration and invasion of SCC9 cells upon DGKG silencing. **K** qRT-PCR analysis indicating downregulation of lipid synthesis genes (ACC1 and FASN) after DGKG knockdown. **L** BODIPY staining showing reduced lipid droplet accumulation in DGKG-silenced SCC9 cells under fluorescence microscopy. **M** Quantification of lipid droplet counts confirming significant reduction

We confirmed the expression of DGKG through analysis of the Human Protein Atlas HPA database. A marked increase in DGKG expression was found in OSCC tissues compared to normal oral mucosal epithelium (Fig. 6A). Following this, we conducted an analysis of DGKG expression at the mRNA and protein levels in normal oral epithelial keratin-forming cell lines and OSCC cell lines utilizing real-time quantitative PCR and Western blot techniques. Consistent with our findings, we noted heightened mRNA expression of DGKG in OSCC cell lines (CAL27, SCC9, and SCC25) compared to control cells (HaCaT) (Fig. 6C), with statistically significant differences in protein expression observed only in the SCC9 cell line (Fig. 6B, D).

### Transfection efficiency

To investigate DGKG's role in OSCC, small interfering RNA (siRNA) targeting DGKG was transfected into SCC9 cells to establish DGKG knockdown lines. After 48 h, Western blot analysis confirmed a significant reduction in DGKG expression in the si-DGKG-1 and si-DGKG-2 groups compared to the si-NC group, with si-DGKG-2 showing the strongest knockdown effect (Fig. 6E). Thus, si-DGKG-2 was selected for further functional experiments.

### Knockdown of DGKG has no significant effect on cell proliferation

In order to examine the impact of DGKG on the proliferative capacity of oral cancer cells, CCK8 assay and colony formation assay were utilized for assessment. The findings from the CCK8 assay indicated that there was no notable alteration in the cell proliferation capability of SCC9 cells at 24 h, 48 h, and 72 h post-transfection with si-DGKG in comparison to the si-NC group (Fig. 6F). Similarly, the results of the colony formation assay demonstrated no significant disparity in the cell colony formation potential of SCC9 cells following transfection with si-DGKG when compared to the control group (Fig. 6G).

### Knockdown of DGKG inhibits cell migration and invasion

Cell migration was assessed using a scratch assay, showing a significant reduction in scratch healing ability at 16 and 32 h in the si-DGKG group compared to the si-NC group (Fig. 6H). Similarly, the transwell assay demonstrated decreased migration potential in SCC9 cells after DGKG knockdown (Fig. 6I). Invasion capability was evaluated using a transwell assay, where 100,000 cells were seeded in migration chambers. Microscopic imaging after 24 h showed that DGKG suppression reduced cell invasion capacity (Fig. 6J).

To further explore whether DGKG may influence tumor progression via mechanisms independent of proliferation, we performed correlation analyses using TCGA expression data. Notably, we observed a positive correlation between DGKG expression and the expression of epithelial–mesenchymal transition (EMT) marker genes (*ρ* = 0.15, *p* = 0.001; Figure S1C), suggesting a potential role for DGKG in EMT-related processes. In contrast, DGKG expression showed no correlation with DNA replication signatures (*ρ* = 0.00, *p* = 0.932; Figure S1D), further supporting that its tumor-promoting effects are unlikely to be mediated through proliferation-related mechanisms. These findings suggest that DGKG may contribute to tumor progression via regulation of EMT rather than directly affecting cell proliferation.

### DGKG knockdown affects lipid metabolism and is associated with key oncogenic signaling pathways

Based on the functional role of DGKG in OSCC, we further investigated its potential involvement in lipid metabolism. Knockdown of DGKG in SCC9 cells led to significantly reduced mRNA expression levels of key lipogenic genes, including ACC1 and FASN, as assessed by qRT-PCR (Fig. 6K). In addition, BODIPY staining revealed a notable decrease in lipid droplet content in DGKG-depleted cells compared to controls, as shown by both fluorescence microscopy and quantitative lipid droplet counts (Fig. 6L, M). These findings suggest that DGKG may play a role in lipid metabolic reprogramming in OSCC.

To further explore the downstream signaling pathways potentially regulated by DGKG, we performed a correlation analysis using transcriptomic data from the TCGA database. A significant positive correlation was found between DGKG expression and the activity of the PI3K/AKT/mTOR pathway (correlation coefficient = 0.176, *P* = 7.28e–05; Figure S1A). Similarly, the TGFB pathway also showed a significant positive correlation with DGKG levels (correlation coefficient = 0.131, *P* = 3.33e–03; Figure S1B). These results imply that DGKG may contribute to OSCC progression via modulation of oncogenic signaling pathways, including lipid metabolism and key regulatory axes such as PI3K/AKT/mTOR and TGFB.

Moreover, correlation analyses using TCGA data revealed a positive association between DGKG expression and EMT marker genes (*ρ* = 0.15, *p* = 0.001, Figure S1C), indicating that DGKG contributes to OSCC progression through EMT-related mechanisms rather than directly influencing proliferative signaling. No significant correlation was found with DNA replication-related signatures (*ρ* = 0.00, *p* = 0.932, Figure S1D). Taken together, these results suggest that DGKG influences tumor growth through mechanisms beyond cell proliferation, including the promotion of EMT, enhancement of migration and invasion, modulation of lipid metabolism, and engagement of oncogenic signaling pathways.

## Discussion

In this study, we conducted a comprehensive untargeted lipidomics analysis combined with machine learning and functional validation to explore lipid metabolic alterations in oral squamous cell carcinoma (OSCC). Our major findings are: (1) OSCC exhibits substantial dysregulation in glycerophospholipid and sphingolipid metabolism; (2) development of a highly accurate lipid-based diagnostic model; and (3) validation of DGKG as a novel risk gene that promotes OSCC cell migration and invasion via lipid metabolic reprogramming. These insights advance our understanding of OSCC biology and suggest promising diagnostic and therapeutic directions.

Altered lipid metabolism is now widely accepted as a hallmark of cancer, supporting processes such as tumor proliferation [36], immune evasion [37, 38], and metastasis [39]. Consistent with this paradigm, our lipidomic analysis revealed prominent disruptions in glycerophospholipid and sphingolipid pathways in OSCC. These findings align with reports from gastric [32], cutaneous squamous [40], and melanoma [41] cancers, where lipid remodeling impacts cell signaling and membrane dynamics. Sphingolipid dysregulation, in particular, plays a critical role in modulating inflammation and survival pathways, and has been implicated in various malignancies, including colorectal [42], breast, and lung cancers [43]. The therapeutic modulation of sphingolipid genes via TRI-Gel [44] underscores the clinical potential of this axis.

Notably, our study extends lipidomics research to OSCC, a cancer type with relatively sparse metabolic characterization. The significant lipid alterations observed support the concept that OSCC hijacks lipid pathways to drive progression. To leverage this diagnostically, we constructed a multivariate SVM model based on 10 lipids, achieving 98.2% accuracy (AUC = 0.996) in training and 95.7% in validation. This performance highlights lipidomics as a minimally invasive, high-performance tool for early OSCC detection. While prior efforts have explored proteomic [45] and general metabolomic markers [46–48], few have focused specifically on lipid profiles despite growing evidence of their relevance [36–38]. Future work should test this model across multi-center cohorts and explore practical implementation through mass spectrometry-based diagnostic kits.

Interestingly, lifestyle factors such as sleep deprivation and physical inactivity were significantly associated with OSCC in our cohort and may intersect with the observed lipidomic alterations. Sleep loss has been shown to disrupt circadian lipid regulation, impair lipolysis, and promote cancer metastasis through pathways such as kynurenic acid signaling [49, 50]. Physical inactivity is also linked to unfavorable lipidomic profiles and tumor-supportive metabolic environments [51, 52]. These findings suggest a mechanistic link between lifestyle behaviors and lipid metabolism in OSCC pathogenesis, highlighting the potential role of behavioral interventions in prevention strategies. These observations also prompt reconsideration of traditional anthropometric indicators, such as BMI, in evaluating metabolic risks related to OSCC. Although previous studies have reported an association between obesity and poor OSCC outcomes [53], our study did not observe significant differences in BMI between OSCC patients and healthy controls. This discrepancy may reflect the limited sensitivity of BMI in capturing cancer-relevant metabolic alterations [54]. Our results suggest that lipid metabolic dysregulation in OSCC may occur independently of BMI, emphasizing the need for molecular markers beyond traditional anthropometric indices.

We also identified DGKG as a potential oncogenic driver. Both transcriptomic and protein analyses confirmed its upregulation in OSCC cells. Functionally, DGKG knockdown significantly impaired OSCC cell migration and invasion, but not proliferation, indicating a primary role in metastatic behavior. DGKG encodes a diacylglycerol kinase that converts DAG to PA—lipids central to membrane synthesis and oncogenic signaling, including the mTOR pathway [55]. In agreement, silencing DGKG reduced ACC1 and FASN expression and lipid dr [54]oplet formation, suggesting a direct role in lipogenesis regulation [25]. The integration of lipidomic, transcriptomic, and functional validation in this study provides a systems-level understanding of OSCC metabolism. From a translational perspective, the 10-lipid diagnostic panel and DGKG represent promising candidates for clinical assay development. Therapeutically, DGKG emerges as a tractable target. While FASN and ACC inhibitors are under active clinical evaluation [56, 57], targeting upstream regulators like DGKG remains underexplored. Inhibitors such as R59022, R59949, and ritanserin—as well as newer compounds like CU-3 and ASP1570—have shown potential to suppress DGK activity, modulate DAG/PA signaling, and enhance immune function [25]. These findings support the rationale for pursuing DGKG-targeted approaches in OSCC.

This study has limitations. The diagnostic model was trained on a single-center cohort, and its generalizability must be validated in larger, diverse datasets. Additionally, while we show that DGKG regulates lipid metabolism and motility, mechanistic studies are needed to elucidate downstream effectors and in vivo relevance. Finally, although this study identified significant associations between lipid metabolism and gene expression in OSCC, the regulatory mechanisms remain unclear.

This study has several limitations. First, the diagnostic model was developed using a single-center cohort, which may limit its generalizability; validation in larger, multi-center populations is warranted. Second, although untargeted lipidomics combined with rigorous statistical modeling supports our findings, the lack of targeted validation may constrain the translational potential of individual biomarkers. Future studies should include targeted assays in independent cohorts. Finally, while we revealed associations between lipid metabolism and gene expression in OSCC and identified DGKG as a potential regulator of lipid-driven motility, the underlying mechanisms and in vivo relevance require further investigation.

## Conlusion

In conclusion, our study highlights the pivotal role of lipid metabolism in oral squamous cell carcinoma (OSCC) and suggests two key translational applications: (1) a lipid-based diagnostic tool for improved accuracy and (2) DGKG as a promising therapeutic target to inhibit tumor progression by modulating lipid metabolic pathways. These findings pave the way for enhanced OSCC diagnosis and treatment strategies. Looking forward, validation of the lipid panel in larger, diverse cohorts is essential to confirm its clinical utility. Additionally, the development of rapid, cost-effective mass spectrometry assays for routine diagnostics is crucial. Future studies should also focus on the mechanistic exploration of DGKG, its downstream effectors, and the efficacy of DGKG inhibitors in preclinical OSCC models. Moreover, integrative analyses using gene–metabolite regulatory networks will help clarify key molecular interactions underlying OSCC lipid dysregulation.

## Supplementary Information

Supplementary Material 1.

Supplementary Material 2.

Supplementary Material 3.

Supplementary Material 4.

## Data Availability

The data and material used to support the findings of this study are available from the corresponding author upon reasonable request. The transcriptomic data analyzed in this study were obtained from The Cancer Genome Atlas (TCGA) database (https://portal.gdc.cancer.gov/). Protein expression data were retrieved from the Human Protein Atlas (HPA) database (https://www.proteinatlas.org/).

## Abbreviations

OSCC: Oral Squamous Cell Carcinoma
UHPLC/Q-Orbitrap HRMS: Ultra-high-performance liquid chromatography quadrupole-Orbitrap high-resolution accurate mass spectrometry
DGKG: Diacylglycerol kinase γ
PC: Phosphatidylcholine
TG: Triglycerides
Cer: Ceramides
SM: Sphingomyelin
LPC: Lyso-phosphatidylcholine
PE: Phosphatidylethanolamine
PG: Phosphatidylglycerol
PCA: Principal component analysis
OPLS-DA: Orthogonal partial least squares discriminant analysis
VIP: Variable importance in projection
ROC: Receiver operating characteristic
AUC: Area under the curve
SVM: Support vector machine
MCCV: Monte-Carlo cross-validation
HMDB: Human Metabolome Database
TCGA: The Cancer Genome Atlas
HPA: Human Protein Atlas
DAG: Diacylglycerol
PA: Phosphatidic acid
ACC1: Acetyl-CoA carboxylase 1
FASN: Fatty acid synthase
PI3K: Phosphoinositide 3-kinase
AKT: Protein kinase B
mTOR: Mechanistic target of rapamycin
TGFB: Transforming growth factor beta
BMI: Body mass index
QC: Quality control
HC: Healthy controls
CI: Confidence intervals
