# Supplementary material for: Lipid metabolites as biomarkers and therapeutic targets in oral squamous cell carcinoma
Source: BMC Oral Health. 2025 Aug 31;25:1390. doi: 10.1186/s12903-025-06700-0 (PMC12400617; doi:10.1186/s12903-025-06700-0)
Supplement: Supplementary file 1 — Supplementary Material 1. [file 12903_2025_6700_MOESM1_ESM.docx]

### Figure S1. Correlation analysis of DGKG expression with key oncogenic pathways in OSCC.

### Positive correlation between DGKG expression and PI3K/AKT/mTOR pathway activity (correlation coefficient = 0.176, P = 7.28e–05).

### (B) Positive correlation between DGKG expression and TGFB pathway activity (correlation coefficient = 0.131, P = 3.33e–03).

### (C) Significant positive association between DGKG expression and epithelial–mesenchymal transition (EMT) marker gene expression (ρ = 0.15, p = 0.001).

### (D) No significant correlation between DGKG expression and DNA replication-related signatures (ρ = 0.00, p = 0.932), suggesting DGKG primarily influences OSCC progression through EMT-related mechanisms.

**
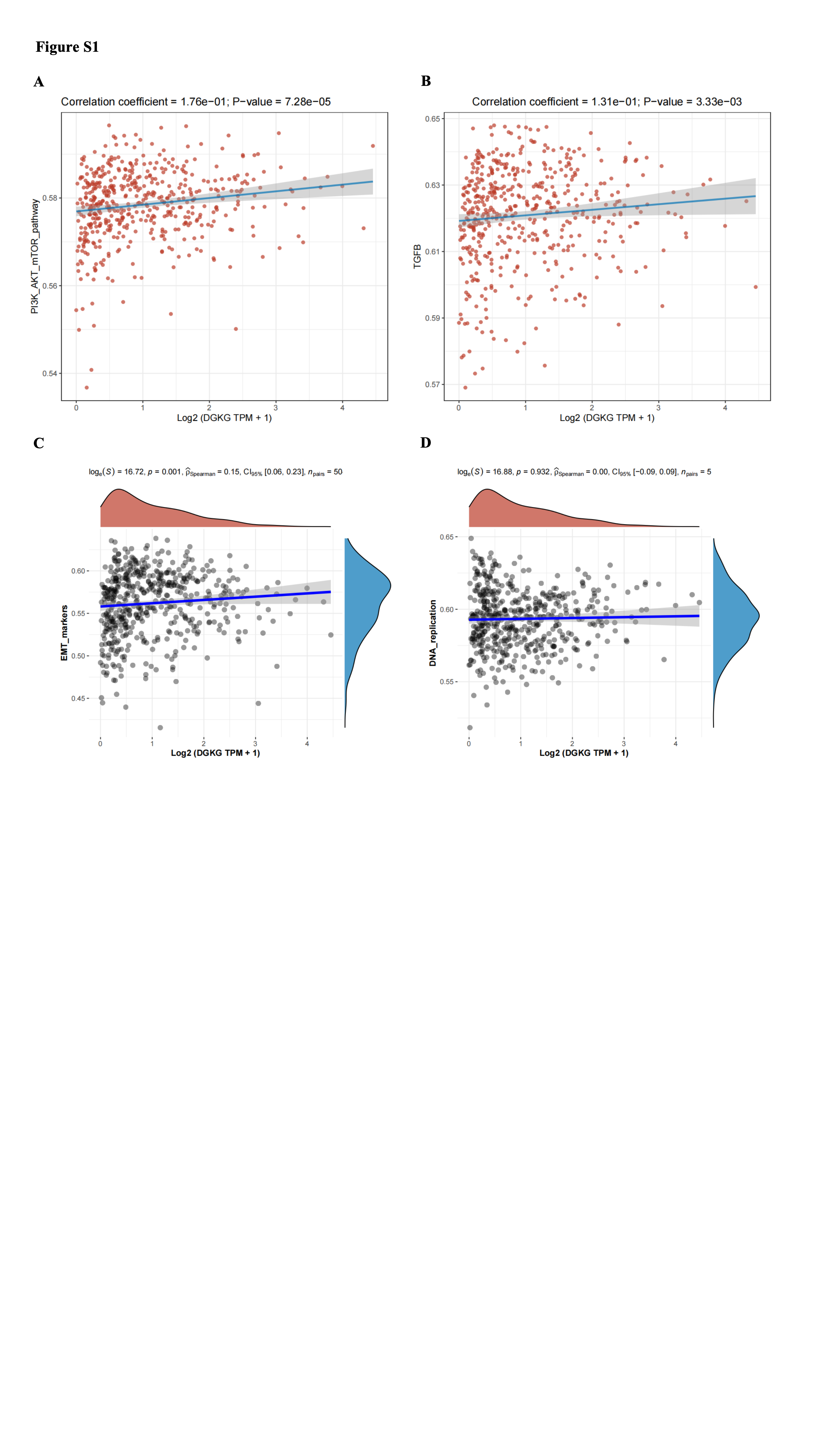
**
