# Supplementary material for: Lipid metabolites as biomarkers and therapeutic targets in oral squamous cell carcinoma
Source: BMC Oral Health. 2025 Aug 31;25:1390. doi: 10.1186/s12903-025-06700-0 (PMC12400617; doi:10.1186/s12903-025-06700-0)
Supplement: Supplementary file 3 — Supplementary Material 3. [file 12903_2025_6700_MOESM3_ESM.docx]

### 2.2.1 Sample pre-processing

Serum lipids were extracted using an MTBE-methanol-water system at -80°C. A mix of 80 µL serum, 600 µL methanol, and 2 mL MTBE was vortexed, followed by the addition of 500 µL water. After centrifugation, the upper organic phase (800 µL) was divided, dried under nitrogen, and reconstituted with isopropanol/acetonitrile for ion assay preparation.

**2.2.2Multivariate ROC and Machine Learning Workflow**

Multivariate ROC curves were generated using the MetaboAnalyst 5.0 platform. Linear support vector machine (SVM) was used as the classification method, and feature selection was performed using the SVM built-in ranking method. Monte-Carlo cross-validation (MCCV) with balanced sub-sampling was applied: in each iteration, 2/3 of the data were used for model training and 1/3 for testing. This process was repeated 100 times. Models were built using the top-ranked 2, 3, 5, 10…up to 100 lipid features. AUC and 95% confidence intervals were calculated to evaluate model performance across iterations.

**2.2.3Lipid droplet staining and quantification**

To assess lipid droplet accumulation, SCC9 cells were seeded into 24-well plates and transfected with siRNA targeting DGKG or a negative control. After 48 hours, cells were washed twice with PBS and fixed in 4% paraformaldehyde for 15 minutes at room temperature. Subsequently, cells were incubated with 2 μM BODIPY 493/503 (MCE，HY-W090090 ) in PBS for 30 minutes in the dark to stain neutral lipids. Nuclei were counterstained with DAPI (1 μg/mL) for 5 minutes. Fluorescence images were captured using a fluorescence microscope under consistent exposure settings.

For quantification, the number of lipid droplets per cell was analyzed using ImageJ software. At least five randomly selected fields per condition were analyzed, and the average lipid droplet count per cell was calculated. Data were expressed as mean ± standard deviation, and statistical significance was determined using a two-tailed Student’s t-test.

**2.2.4 Correlation analysis between DGKG expression and oncogenic signaling pathways**

Transcriptomic STAR-counts data and corresponding clinical information for HNSC cancer were downloaded from the TCGA database (https://portal.gdc.cancer.gov). TPM (Transcripts Per Million) values were extracted and subsequently normalized using log2(TPM + 1) transformation. Gene sets corresponding to specific signaling pathways were collected, and single-sample gene set enrichment analysis (ssGSEA) was performed using the R package GSVA, with the parameter set to method = "ssgsea". The correlation between gene expression and pathway activity scores was then assessed using Spearman correlation analysis. All statistical analyses were conducted using R software (version 4.0.3). A p-value of less than 0.05 was considered statistically significant.
