# Supplementary material for: Lipid metabolites as biomarkers and therapeutic targets in oral squamous cell carcinoma
Source: BMC Oral Health. 2025 Aug 31;25:1390. doi: 10.1186/s12903-025-06700-0 (PMC12400617; doi:10.1186/s12903-025-06700-0)
Supplement: Supplementary file 4 — Supplementary Material 4. [file 12903_2025_6700_MOESM4_ESM.docx]

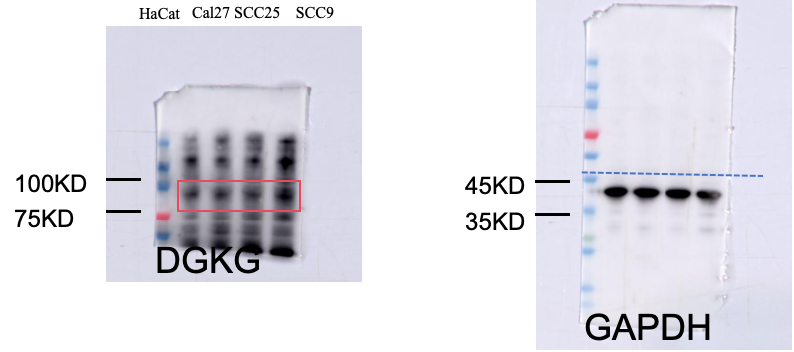


Figure 6B. DGKG protein expression analysis in OSCC cell lines by Western blot.

Representative images from three independent biological replicates performed

under identical conditions are displayed. Cell lines: HaCaT (normal oral

keratinocytes), Cal27, SCC25, SCC9 (OSCC cell lines).


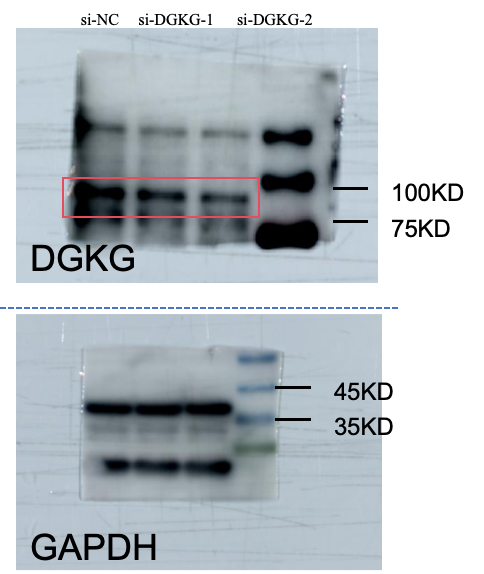


Figure 6E. Validation of DGKG knockdown efficiency in SCC9 cells using siRNA transfection. Representative Western blot from three independent experiments is shown. Cells were transfected with si-NC (negative control), si-DGKG-1, or si-DGKG-2 for 48 hours. si-DGKG-2 demonstrated superior knockdown efficiency and was selected for subsequent functional assays.
